# “My son was castrated as a result of a medical error. Is it OK to raise him as a eunuch?”

**DOI:** 10.1016/j.amsu.2021.102586

**Published:** 2021-07-26

**Authors:** Thomas W. Johnson, Richard J. Wassersug

**Affiliations:** a Department of Anthropology (Emeritus), California State University–Chico, 1537 Kjell Court, Santa Rosa, CA, 95405, USA; b Department of Cellular & Physiological Sciences, University of British Columbia, 2350 Health Sciences Mall, Vancouver, British Columbia, V6T 1Z3, Canada

**Keywords:** Castration, Testicular torsion, Testosterone replacement, Delayed puberty, Eunuch

## Abstract

A 12-year-old boy lost both testes after testicular torsion. He is now 14, and his father wants to know if the boy should immediately start supplemental testosterone or if he might reasonably choose to live as a eunuch. The boy does not yet express any strong opinion except that he is embarrassed about his weight gain. We advised the father that there is no need to rush the decision as the boy could at least delay testosterone therapy until his teens or early 20s and still go through male puberty with little risk of adverse health effects. We seek to know if others endorse our endocrinological advice. The boy's father wants to be honest with his son about the social challenges the boy may face if he elects to delay or avoid puberty altogether and chooses to openly identify as a eunuch.

## Highlights

• A 12-year-old lost both testes following a misdiagnosed bilateral torsion.
• His father wants to know if he must take supplemental testosterone.
• His father wants to support him if he chooses to openly present as a eunuch.

## 1 Introduction

One of us received an email from a father whose son had lost both testicles at age 12 due to medical error. The father wanted advice on how to support his son, now 14, if the son remains comfortable with his hypogonadal state. He specifically asked for our opinion on raising his son as a eunuch. The alternative would be for his son to begin testosterone replacement therapy, which is the standard medical treatment for agonadal individuals who are not gender dysphoric.

## 2 Background

At age 12 a boy experienced bilateral testicular torsion that was misdiagnosed as a severe infection. He was treated ineffectively with antibiotics. By the time he was correctly diagnosed, the ischemia had led to testicular necrosis and his testes could not be saved.

Unilateral testicular torsion, which occurs most frequently during puberty, affects about one in 4000 boys. Bilateral torsion is much rarer, making the diagnosis difficult [1]. Strangulation of the blood supply is a medical emergency. There is virtually no viability of the testicle if surgical intervention is delayed more than 12 h [2]. The boy was properly diagnosed only after 48 h.

The father expressed two concerns: First he wanted assurance that it would be safe for his son to be raised without supplemental testosterone. He understood that his son would not go through puberty without hormonal treatment. In that regard, he sought assurance that the son's health would not be jeopardized in the long term, if he did not begin supplemental testosterone immediately.

The father's other concern was how to support his son socially and emotionally if the son openly presented as a eunuch. The father knew that prepubescent boys in history, in various cultures and at various times, had their testicles removed to live as eunuchs. In those many cultures they were a visibly distinct gender. He also knew that in those societies the eunuchs filled special social roles and could achieve high status. During the castrati period in Italy, for instance, they were celebrated for their uniquely powerful and high-pitched voices. In earlier times, such as during the Byzantine Empire, they were government administrators, diplomats, and occasionally senior military officials. They filled both governmental and military roles in the Ottoman and various other Islamic Empires as well. At times in Chinese history, even prominent families would castrate a young son, who was then eligible to serve in the government. Such families hoped that their sons might rise in the palace bureaucracy and be able to provide support for the family.

In terms of health, we advised the father that the boy should be under the care of a pediatric endocrinologist. We also pointed out the risk of osteoporosis in hypogonadal individuals and the need for adequate Vitamin D and calcium in the boy's diet to protect bone mineral integrity [3].

We asked if the boy was active in any sports that involved running or jumping as impact loading has been shown to be protective against osteoporosis, which is a risk for hypogonadal adults [3]. We were told, however, that he was neither involved in such sports nor motivated to take them up.

The father pointed out that the boy is embarrassed over his recent weight gain and that his classmates recognize his different appearance, where few boys are similarly overweight. We recommended both watching his diet and encouraging exercise. But when asked if the child was bullied by his schoolmates, he said “no”. The boy is reportedly also doing well academically.

The family lives in an area where there is little or no access to appropriate counseling services and they will primarily need to rely on the Internet for advice.

### 2.1 Medical considerations

We encouraged the father to have his son get an endocrinological consult, which he did. That included a blood draw to assay testosterone, lutenizing hormone, follicle stimulating hormone, and estradiol. The profile was consistent with primary hypogonadism: extremely high LH and FSH, elevated estradiol, and very low testosterone.

The endocrinologist warned against the “‘disadvantages’ of never experiencing sexuality and physical unmaturity,” as well as “risks for some cardiovascular diseases and prostate-related diseases,” and recommended initiation of testosterone therapy.

In contrast, the literature suggests that puberty can be safely delayed at least until the boy is the age of majority. There are various case reports of anorchia diagnosed in adulthood in the medical literature and they suggest that individuals treated with testosterone beginning in their late teens and twenties completed normal puberty [4,5]. We informed the father that data are few, but one report from Korea suggests that eunuchs lived longer than eugonadal males [6]. Lower testosterone is associated with lower risk for prostate cancer, malignant melanoma, and possibly liver cancer [7]. Androgen deprivation however is associated with increased risk of cardiovascular disease and diabetes in older men treated with LHRH agonist drugs for prostate cancer [8]. For older men, lifestyle interventions that prevent metabolic syndrome may mitigate those risks, but the impact on younger men is unexamined. Androgen deprivation not only reduces the risk of prostate cancer but is the primary treatment for systemic prostate cancer [9].

### 2.2 Social considerations

Although eunuchs have been the most common and consistent non-binary gender throughout history, they are absent from the contemporary expansion of genders in the western world [10]. There are various reasons for why they are uncommon and/or invisible [11,12]. Among these reasons is that, since the 20th century, anorchic males and those castrated pre-puberty often have access to supplemental testosterone, in which case they do eventually go through male puberty and will not be readily identifiable in public as having had a genital ablation. For them, administering testosterone is often a straightforward choice, especially as there are no longer prestigious “eunuch” roles to which a castrated individual might realistically aspire. Conversely, the label “eunuch” is now commonly used metaphorically and pejoratively to indicate a globally powerless individual [13] suggesting substantial stigma associated with that gender identity.

Castration of prepubescent males to make them eunuchs is now seen as unethical and barbaric. In cases like the one here, where a boy was castrated as a result of inadequate medical care, the standard follow-on of care is to start the boy promptly on testosterone, which encourages normal progression through puberty. The argument for doing so is that without supplemental testosterone the boy would not acquire male secondary sexual characteristics. He would look different and sound different from other males his age. He would grow taller, have proportionately more body fat, and retain a high-pitched voice. He might then be subject to ridicule for his unusual appearance.

The father (and presumably the son to the extent that he can as a child) recognize that open identification as a eunuch might exacerbate stigma. The father understands that label is now largely used metaphorically with negative connotations. Despite history that contradicts the stereotype, today “eunuch” implies that someone is an inferior male in a gender hierarchy [14].

The questions then are: With increasing acceptance in the western world of gender presentations that are variously called non-binary, gender queer, and androgynous, can the eunuch gender be resurrected? Can this be done in a way that does not lead to discrimination against a genetic male, who in fact lacks testes, is androgen deficient since childhood, and elects to identify as a “eunuch”?

Although there are various medical conditions that lead to testosterone deficiency in adult males, we know of no cases in the modern world where a male, who lost his testes before puberty, openly identified his gender as eunuch. There has been, however, a somewhat similar case of a genetic female, who was put on puberty blocking drugs at age 11 to treat severe gender dysphoria [15]. That individual wished to stay on gonadal suppressing drugs without any supplemental gonadal hormones. Unlike a hypogonadal male, however, as an adult a hypogonadal female would still appear female, though with minimal breast development and narrower hips than most other females of similar age.

## 3 Conclusion

Of all the challenges to a gender binary, there is probably none with as strong a historical precedent than the eunuch. In the absence of any current evidence of medical or social risk, we suggested to the father that his son could safely wait until his late teens or early 20s to make a decision as an adult about whether he wished to take supplemental testosterone or live his life as a eunuch.

The father read and approved a draft of this essay. We plan to share all responses with him. We invite input from ethicists, endocrinologists, and other health care providers on what the father can do to help support his son in whatever decision the son makes.

## Ethical approval

No approval was sought or received.

## Consent

We received permission from the boy's father via email.

## Author contribution

Both authors contributed equally.

## Funding sources

No funding was received.

## Registration of research studies

Name of the registry: **N/A**
Unique Identifying number or registration ID: **N/A**
Hyperlink to your specific registration (must be publicly accessible and will be checked): **N/A**

## Guarantor

We, the authors, Thomas W. Johnson and Richard J. Wassersug, are the guarantors.

## Declaration of competing interest

Nothing to declare.
